# Supplementary material for: Empirical methods for controlling false positives and estimating confidence in ChIP-Seq peaks
Source: BMC Bioinformatics. 2008 Dec 5;9:523. doi: 10.1186/1471-2105-9-523 (PMC2628906; doi:10.1186/1471-2105-9-523)
Supplement: Additional File 1 — A variety of html documents from the USeq web site detailing the available applications, their best usage, output file type descriptions, command line menus, etc. [file 1471-2105-9-523-S1.zip › USeqUserGuides/JavaDoc.zip!/JavaDoc/bioroot/class-use/ReagentBean.html]

Uses of Class bioroot.ReagentBean


|  |  |  |
| --- | --- | --- |
| **Overview** \| **Package** \| **Class** \| **Use** \| **Tree** \| **Deprecated** \| **Index** \| **Help** | |  |
| PREV   NEXT | **FRAMES**    **NO FRAMES**     **All Classes** |  |


---


## **Uses of Class bioroot.ReagentBean**

| Packages that use ReagentBean | |
| --- | --- |
| **bioroot** |  |
| **bioroot.antibody** |  |
| **bioroot.oligo** |  |
| **bioroot.plasmid** |  |
| **bioroot.strain** |  |

Uses of ReagentBean in bioroot

| Methods in bioroot with parameters of type ReagentBean | |
| --- | --- |
| `static java.lang.String` | `Util.getViewLine(ReagentBean[] reagentBeans, int[] inView, Preference reagentPreference, java.lang.String reagentType)`             Returns hot linked html for 1-30 31-60 ... |
| `boolean` | `DBUtil.updateCommonAccessFields(ReagentBean reagentBean, java.lang.String tableName)`             Just updates fields that are accessible by all lab group memebers. |
| `boolean` | `DBUtil.updateCommonAccessFields(ReagentBean bean, UserBean userBean, javax.servlet.http.HttpServletRequest request)`             Just update the commonly accessible fields: lastUser, comments |

Uses of ReagentBean in bioroot.antibody

| Subclasses of ReagentBean in bioroot.antibody | |
| --- | --- |
| `class` | `AntibodyBean` |

Uses of ReagentBean in bioroot.oligo

| Subclasses of ReagentBean in bioroot.oligo | |
| --- | --- |
| `class` | `OligoBean` |

Uses of ReagentBean in bioroot.plasmid

| Subclasses of ReagentBean in bioroot.plasmid | |
| --- | --- |
| `class` | `PlasmidBean` |

Uses of ReagentBean in bioroot.strain

| Subclasses of ReagentBean in bioroot.strain | |
| --- | --- |
| `class` | `StrainBean` |

---


|  |  |  |
| --- | --- | --- |
| **Overview** \| **Package** \| **Class** \| **Use** \| **Tree** \| **Deprecated** \| **Index** \| **Help** | |  |
| PREV   NEXT | **FRAMES**    **NO FRAMES**     **All Classes** |  |


---
